# Membrane Integrity Contributes to Resistance of Cryptococcus neoformans to the Cell Wall Inhibitor Caspofungin

**DOI:** 10.1128/msphere.00134-22

**Published:** 2022-06-27

**Authors:** Brenda Moreira-Walsh, Abigail Ragsdale, Woei Lam, Rajendra Upadhya, Evan Xu, Jennifer K. Lodge, Maureen J. Donlin

**Affiliations:** a Edward A. Doisy Department of Biochemistry and Molecular Biology, Saint Louis University School of Medicine, St. Louis, Missouri, USA; b Department of Molecular Microbiology, Washington University School of Medicine, St. Louis, Missouri, USA; Duke University Medical Center

**Keywords:** antifungal agents, antifungal resistance, *Cryptococcus neoformans*, fungal cell wall

## Abstract

The fungal pathogen Cryptococcus neoformans causes up to 278 000 infections each year globally, resulting in up to 180,000 deaths annually, mostly impacting immunocompromised people. Therapeutic options for C. neoformans infections are very limited. Caspofungin, a member of the echinocandin class of antifungals, is generally well tolerated but clinically ineffective against C. neoformans. We sought to identify biological processes that can be targeted to render the cell more susceptible to echinocandins by screening the available libraries of gene deletion mutants made in the KN99α background for caspofungin sensitivity. We adapted a Candida albicans fungal biofilm assay for the growth characteristics of C. neoformans and systematically screened 4,030 individual gene deletion mutants in triplicate plate assays. We identified 25 strains that showed caspofungin sensitivity. We followed up with a dose dependence assay, and 17 of the 25 were confirmed sensitive, 5 of which were also sensitive in an agar plate assay. We made new deletion mutant strains for four of these genes: *CFT1*, encoding an iron transporter; *ERG4*, encoding a sterol desaturase; *MYO1*, encoding a myosin heavy chain; and *YSP2*, encoding a sterol transporter. All were more sensitive to membrane stress and showed significantly increased sensitivity to caspofungin at higher temperatures. Surprisingly, none showed any obvious cell wall defects such as would be expected for caspofungin-sensitive strains. Our microscopy analyses suggested that loss of membrane integrity contributed to the caspofungin sensitivity, either by allowing more caspofungin to enter or remain in the cell or by altering the location or orientation of the enzyme target to render it more susceptible to inhibition.

**IMPORTANCE** The intrinsic resistance of Cryptococcus neoformans to the cell wall inhibitor caspofungin limits the available therapies for treating cryptococcal infections. We screened a collection of more than 4,000 gene deletion strains for altered caspofungin sensitivity to identify biological processes that could be targeted to render the cell more susceptible to caspofungin. We identified multiple genes with an effect on caspofungin susceptibility and found that they were associated with altered membrane permeability rather than the expected cell wall defects. This suggests that targeting these genes or other genes affecting membrane permeability is a viable path for developing novel therapies for treating this global fungal pathogen.

## INTRODUCTION

Cryptococcus neoformans is a fungal pathogen of immunocompromised people that causes an estimated 278,000 infections each year among HIV-positive patients globally, resulting in up to 181,000 deaths annually (1). C. neoformans infections can be successfully treated with amphotericin B (AMB) and 5-flucytosine or fluconazole (FLC), but the therapy regimens are long and the drugs have significant toxicity. The mortality from cryptococcal infections remains 15 to 30% even in the context of antiviral treatments for HIV (2–4). Members of the echinocandin class of antifungal drugs inhibit the synthesis of β-glucan in the fungal cell wall by targeting the essential gene *FKS1*, which encodes the enzyme β-1,3-glucan synthase. The echinocandins are typically well tolerated because they target a fungus-specific protein. One echinocandin, caspofungin, can inhibit the C. neoformans β-1,3-glucan synthase, but not at a therapeutically useful concentration (5). We and others have explored the question of how C. neoformans is so resistant to this well-tolerated class of antifungals (6–8). Due to the inherent resistance of C. neoformans to caspofungin, identifying genes or pathways that can be targeted to render the cell more susceptible to this class of antifungals is a potential strategy for the development of new combination therapies.

The resistance of C. neoformans to echinocandins is modulated by a combination of stress-activated processes. Deletion mutants with deletions of the mitogen-activated protein (MAP)-type kinases in the cell wall integrity (CWI) pathway show increased sensitivity to a variety of cell wall stressors but only modestly increased sensitivity to caspofungin (6). However, deleting the Pkc1 kinase in the CWI pathway results in severe cell wall defects and a much lower MIC for caspofungin (unpublished observations; 9). Studies in the past few years have identified a beta-subunit of a lipid translocase, encoded by *CDC50* (CNAG_06465), and a dihydroorotate dehydrogenase (DHOH), encoded by *URA1* (CNAG_02794), that both play a role in resistance to caspofungin (8, 10). More recently, the Cdc50 protein was proposed to interact with a calcium channel protein to regulate calcium homeostasis, whereas the DHOH enzyme is required for *de novo* pyrimidine synthesis (11). Two studies have previously conducted systematic screening of the available gene deletion mutant libraries for caspofungin sensitivity. Huang and coworkers screened a library of ~3,300 single-gene-deletion mutants and identified two ergosterol biosynthetic genes that, when deleted, increased caspofungin sensitivity, but they did not conduct any further characterization of those two deletion mutant strains (8). More recently, Pianalto and coworkers conducted a systematic screen of that same library using a different medium and identified 14 genetic mutants with increased caspofungin sensitivity. Based on the identity and known functions of the mutated genes, they proposed that disruption of other stress-activated pathways results in caspofungin sensitivity (7). We know that the stress-activated pathways work in tandem and that disruption of one pathway can activate or impair a second pathway. For example, we observed that deletions of kinases in the CWI pathway alter cAMP levels, indicating cross talk with the cAMP/protein kinase A (PKA) pathway, which is also known to have a role in cell wall integrity (12, 13). Our motivation for this work was to expand the identification of genes and processes with a role in caspofungin resistance by systematically screening for altered caspofungin sensitivity the ~4,000 gene deletion mutants made in the KN99α background that are available in two libraries from the Fungal Genetics Stock Center (14).

## RESULTS

### Screening the UCSF 2015 and 2016 deletion mutant libraries for caspofungin sensitivity.

We developed a screening assay to identify C. neoformans gene deletion strains with altered caspofungin sensitivity using the following criteria: (i) the assay could be conducted in liquid culture in a 96-well plate format, (ii) would be reproducible, (iii) could correct for different growth rates, so that slow-growing strains would not be identified as sensitive, and (iv) would use a metabolic readout to eliminate the variability of optical density readings. We modified a fungal biofilm assay developed for screening Candida albicans to reflect the growth characteristics of C. neoformans (15). We used the defined medium yeast nitrogen base (YNB) with 2% glucose (YNB 2%) at pH 7 as our medium to eliminate the variability of yeast extract-peptone-dextrose (YPD) formulations and conducted the assay at 25°C to capture strains that might show temperature sensitivity unrelated to altered sensitivity to caspofungin. The screening assay was developed using deletion strains with known sensitivity to caspofungin to calibrate the assay and to determine the optimal concentration of caspofungin to uncover phenotypes (Fig. S1 in the supplemental material). We screened the 4,030 deletion strains from the UCSF 2015 and 2016 deletion libraries (14). The libraries were obtained from the Fungal Genetics Stock Center and aliquoted into 41 96-well plates.

10.1128/msphere.00134-22.1 FIG S1 Dose dependence of caspofungin in 17 deletion strains identified by screening as sensitive to caspofungin. Overnight cultures of cells were washed with YNB and diluted to an OD_650_ of 0.001 before caspofungin was added at final concentrations of 0, 10, 15, 20, and 30 μg/mL to 3 replicates. The cells were incubated for 48 h at 25°C. XTT reagent was added to all wells, the plates incubated for 3 h at 37°C and then centrifuged, and 100-μL amounts transferred to fresh plates. The absorbance was read at 450 nm, and the data normalized by the results for the untreated control for each strain. Data are presented as percentages of the results for untreated cells and as mean values with error bars showing the standard deviations. Download FIG S1, EPS file, 0.7 MB. Copyright © 2022 Moreira-Walsh et al. 2022 Moreira-Walsh et al. https://creativecommons.org/licenses/by/4.0/ This content is distributed under the terms of the Creative Commons Attribution 4.0 International license.

**FIG 1:**
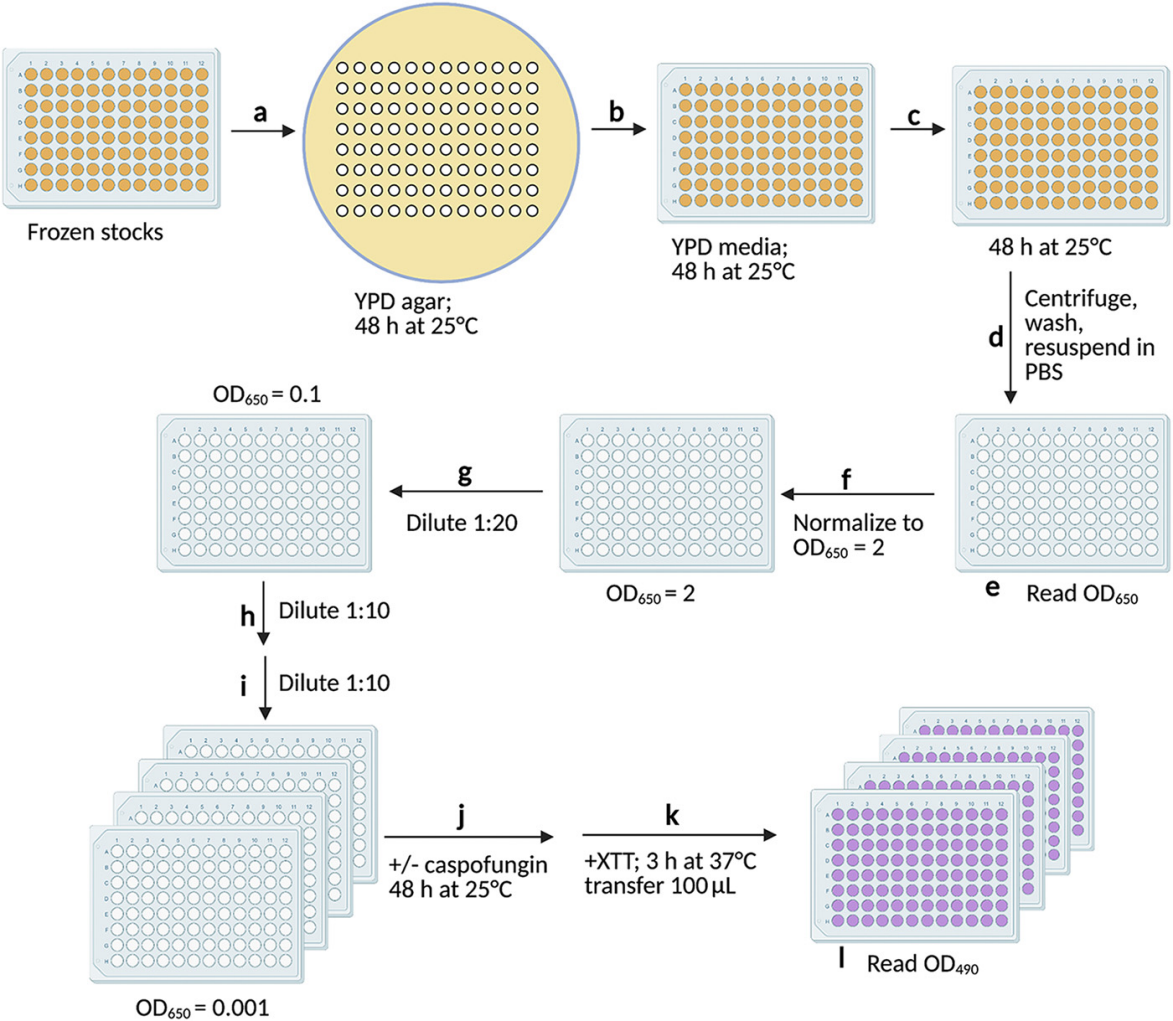
Schematic of the caspofungin sensitivity screening assay. Steps and conditions: (a) frozen cells were transferred to YPD agar using a pronged replicator and incubated for 48 h at 25°C; (b) cells were transferred to 600 μL YPD in a deep-well plate using a multichannel pipettor and incubated for 48 h at 25°C; (c) 30 μL was transferred to 600 μL YPD in a deep-well plate and incubated for 48 h at 25°C; (d) cells were centrifuged, washed 2 times, and resuspended in 200 μL PBS; (e) cells were transferred to a clear, flat-bottom plate and optical densities measured at 650 nm; (f) cells were normalized to an OD_650_ of 2 in a final volume of 120 μL PBS; (g) cells were diluted 1:20 in 200 μL YNB; (h) cells were diluted 1:10 in 100 μL YNB; (i) cells were diluted 1:10 in 100 μL YNB in 4 replicate plates; (j) caspofungin was added at 10 μg/mL to 3 replicates, PBS was added to the control plate, and the plates were incubated for 48 h at 25°C; (k) XTT reagent was added to all plates, the plates incubated for 3 h at 37°C and centrifuged, and 100 μL transferred to fresh plates; and (l) absorbance was read at OD_490_. Plates incubated in the 2nd, 3rd, and 10th steps were covered with a Breathe-Easy membrane. Image created with BioRender.com.

**FIG 2:**
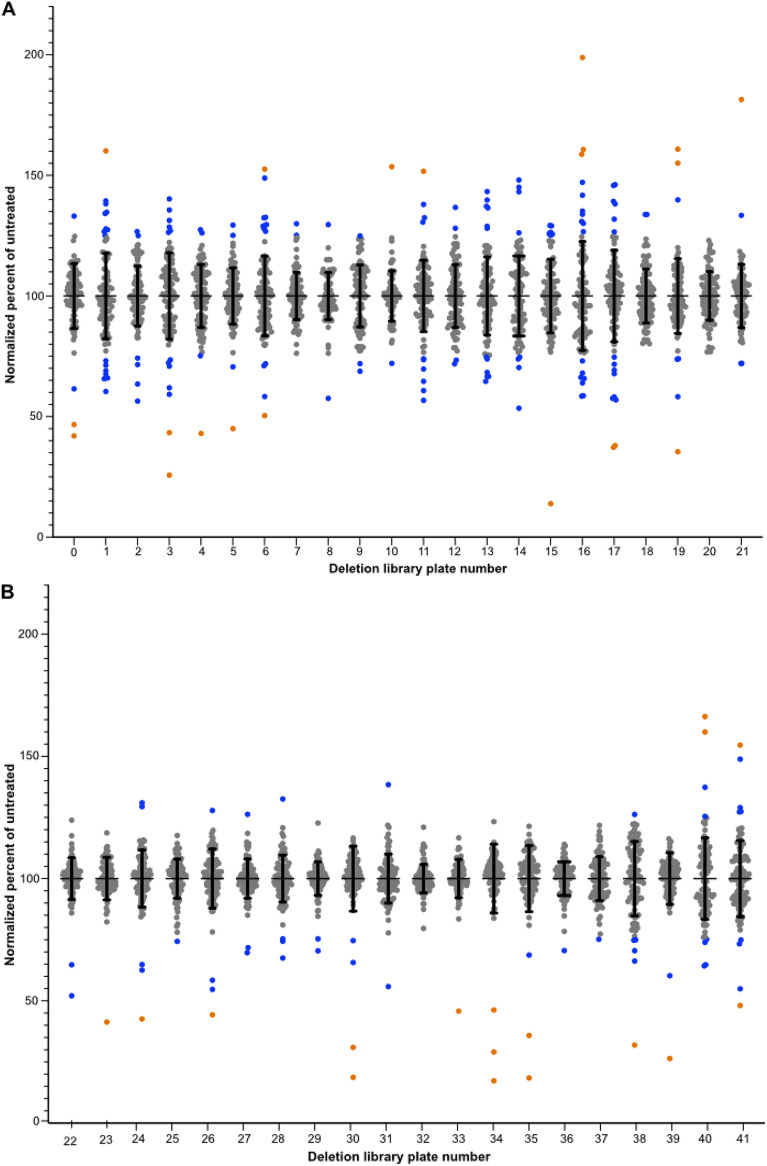
Caspofungin sensitivity screening data for all deletion strains. Normalized growth of each strain on a given plate relative to the untreated cells is shown for the 2015 library (A) and the 2016 library (B) strains. Each dot represents the average percentage of growth relative to the growth of untreated cells after normalization across all strains in the same plate. Error bars show standard deviations. Orange circles represent strains with percent inhibition values of ±2 standard deviations from the plate average. Blue circles are strains with percent inhibition values of ±1 standard deviation from the plate average.

The schematic of the screening assay is depicted in Fig. 1, and the supplemental material contains a step-by-step protocol for the screening assay. The essential parameters for this assay were that each plate was replicated directly from the frozen 96-well plates in the library and passaged the same number of times and each strain on a plate was normalized to the same cell density prior to conducting the assay in triplicate at 25°C to eliminate false positives from strains that were temperature sensitive. We employed a metabolic readout to measure the percentages of inhibition compared to the results for the untreated control, which reduced the inherent variability of an optical density assay, particularly with deletion mutant strains whose cells tend to clump. The data were analyzed in Prism and the output calculated as the percentages of the results for untreated cells averaged across the three treated replicates. Sensitive strains were defined as showing 50% or more inhibition of XTT [2,3-bis-(2-methoxy-4-nitro-5-sulfophenyl)-2H-tetrazolium-5-carboxanilide salt] activity relative to the mean of the normalized output across the entire plate. Of the 4,030 deletion strains screened, 25 strains showed increased sensitivity to 10 μg/mL caspofungin under these assay conditions (Fig. 2).

### Confirmation of echinocandin sensitivity phenotypes.

**FIG 3:**
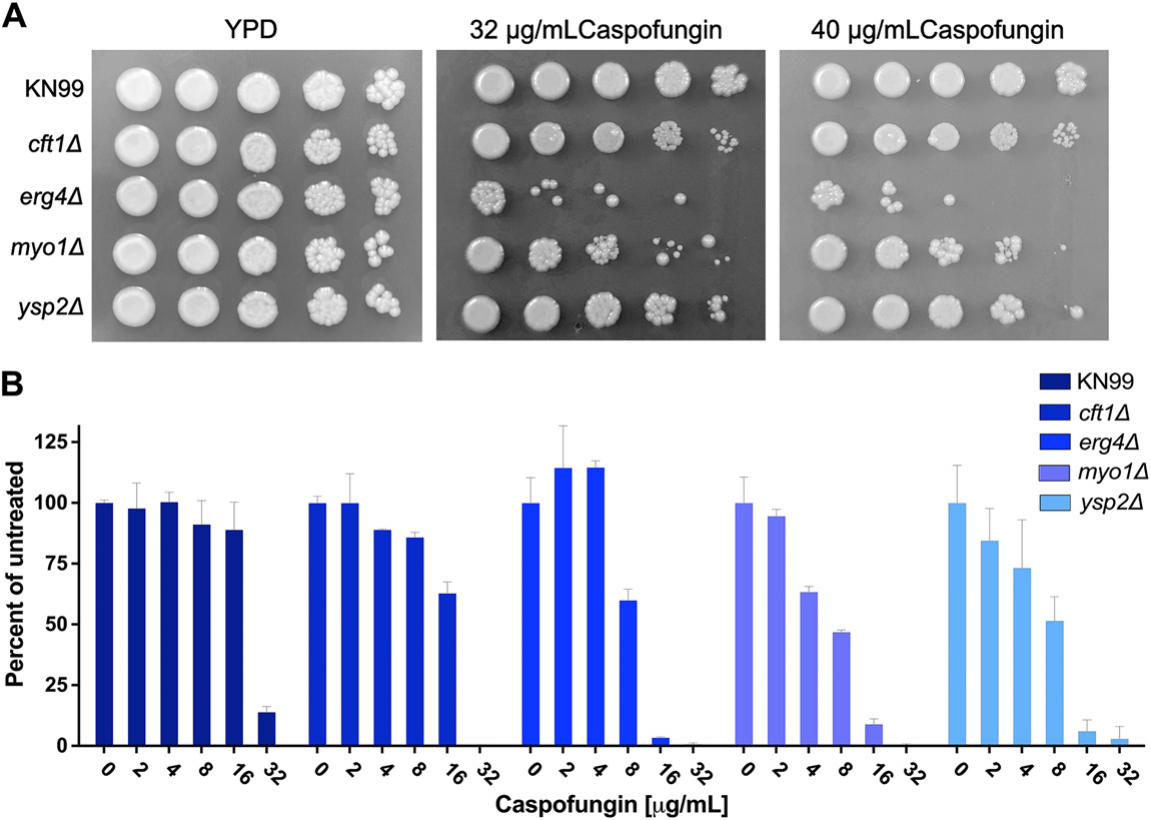
The caspofungin sensitivity was replicated in the new deletion strains. We tested KN99 and the *cft1*Δ, *erg4*Δ, *myo1*Δ, and *ysp2*Δ deletion strains for sensitivity to caspofungin by measuring the MICs of caspofungin in YNB using a microdilution assay (A) and on agar plates (B). Cells at an OD of 0.001 were incubated with caspofungin at concentrations of 2 to 32 μg/mL for 48 h at 30°C. Cell growth was measured at OD_650_ and plotted as the percentage of the growth of the untreated control. Error bars represent the standard deviation of the average of three replicates.

We tested all 25 strains in a dose dependence assay using caspofungin concentrations of 10 μg/mL to 30 μg/mL. Seventeen of the strains were confirmed to show >50% reduction in XTT activity compared to that of the KN99 wild type (WT) at 10 μg/mL caspofungin (Table S1, Fig. S1). All 25 deletion strains showed sensitivity at 20 mg/mL caspofungin, but 8 strains did not meet our criteria of at least 50% inhibition at 10 mg/mL caspofungin. The dose dependence confirmation assay was done using cells that had undergone at least one additional passage on YPD plates relative to the number of passages for the strains used in the screening assay. We tested the 17 strains on solid medium by using a spot assay on YNB agar (pH 7) containing 32 μg/mL caspofungin. Of the 17 strains tested, only 5 showed a consistent sensitivity to caspofungin under these conditions. The difference in sensitivity may be due to the reduced metabolism of cells grown on solid agar or because the yeast cells can grow on top of a layer of dead cells, which may reduce the exposure to the caspofungin in the medium. The genes deleted in the five caspofungin-sensitive deletion strains (Table 1) were *CFT1* (CNAG_06242), encoding a high-affinity iron transporter (16), *ERG4* (CNAG_02830), encoding a sterol desaturase, *MYO1* (CNAG_01536), encoding a myosin heavy chain, *PEP5* (CNAG_06376), with a putative role in vesicle transport, and *YSP2* (CNAG_00650), with a putative role in sterol transport (17). We attempted to create new deletion mutant strains for all five genes in the KN99 parental strain so that we could confirm their caspofungin phenotypes with a second, independent replicate. We successfully made one or more independent strains with deletions of the *CFT1*, *ERG4*, *MYO1*, and *YSP2* genes and used these new strains to confirm their caspofungin sensitivity and explore their phenotypes in response to other cell wall stressors. However, repeated attempts to create a new *PEP5* deletion mutant failed, suggesting that this gene may be essential. We did not attempt to confirm that the *pep5*Δ strain from the library was either a single deletion mutant or had secondary mutations that compensated for the loss of *PEP5.* All four newly made deletion mutants were more sensitive than the wild type to caspofungin, with the *myo1*Δ and *erg4*Δ strains demonstrating the greatest sensitivity in a dose-dependent liquid assay (Fig. 3A) and on agar plates (Fig. 3B) when tested at 30°C. The more subtle phenotype of the *cft1*Δ and *yps2*Δ strains may reflect the reduced metabolic activity when grown on solid medium, which may alter their sensitivity. We also confirmed the caspofungin phenotypes on agar plates using independent isolates (Fig. S2). We also tested the sensitivity of these strains to two other echinocandins, anidulafungin and micafungin. We observed no inhibition by micafungin at concentrations up to 100 μM. The MIC for anidulafungin was 75 μM for KN99 and the *cft1*Δ strain but 37.5 μM for the *erg4*Δ and *myo1*Δ strains, suggesting that the cell wall of these two deletion mutants might have been more accessible to the echinocandins (data not shown). We conducted this experiment prior to obtaining a confirmed *ysp2*Δ strain.

**TABLE 1:** Five deletion strains with confirmed caspofungin sensitivity

Plate	Accession no.^*a*^	Gene	Protein description
0-B12	CNAG_06242	*CFT1*	High-affinity ferric transporter
5-F6	CNAG_02830	*ERG4*	Delta24(24(1))-sterol reductase; ergosterol biosynthesis
23-C7	CNAG_01536	*MYO1*	Myosin heavy chain
34-F12	CNAG_00650	*YSP2*	StART family protein involved in sterol transport
38-C7	CNAG_06376	*PEP5*	Vacuolar protein sorting and E3 ubiquitin ligase

a H99 accession number from FungiDB.

10.1128/msphere.00134-22.2 FIG S2 KN99 and two independent isolates of the *cft1*Δ, *erg4*Δ, *myo1*Δ, and *ysp2*Δ strains were grown overnight in YPD and diluted to an OD_650_ of 1 before being spotted in 4-step serial dilutions on YPD agar plates containing 32 or 40 μg/mL caspofungin. The plates were incubated for 3 days at 30°C (A) or 37°C (B). Download FIG S2, TIF file, 1.8 MB. Copyright © 2022 Moreira-Walsh et al. 2022 Moreira-Walsh et al. https://creativecommons.org/licenses/by/4.0/ This content is distributed under the terms of the Creative Commons Attribution 4.0 International license.

### Stress and antifungal phenotypes of the new deletion mutant strains.

We hypothesized that deletion mutant strains with increased sensitivity to caspofungin might also show increased sensitivity to other cell stresses, as well as other antifungal drugs. We tested sensitivity to fluconazole (FLC) and amphotericin B (AMB) using a limiting dilution assay (Fig. S3). Using a criterion of 50% inhibition compared to the results for the vehicle control, two independent isolates of the *cft1*Δ and *myo1*Δ strains both had the same MIC for FLC as wild-type KN99 (Fig. S3A). The *erg4*Δ isolates had a much lower MIC for FLC (3.13 μM versus 12.5 μM), whereas the *ysp2*Δ isolate had a slightly lower MIC of 6.25 μM. FLC also appears to be fungicidal in the *erg4*Δ strain, although that was not confirmed in a time-kill assay. Two of the deletion mutants, the *myo1*Δ and *ysp2*Δ isolates, had lower MICs for AMB, suggesting an altered or compromised plasma membrane, with the *ysp2*Δ mutant showing sensitivity at the lowest concentration tested (Fig. S3B).

10.1128/msphere.00134-22.3 FIG S3 MICs of fluconazole (A) and amphotericin B (B) in the deletion strains and wild-type KN99. Overnight cultures were diluted to an OD of 0.001 in YNB, pH 7. Plates were incubated at 30°C for 2 days. Download FIG S3, EPS file, 0.5 MB. Copyright © 2022 Moreira-Walsh et al. 2022 Moreira-Walsh et al. https://creativecommons.org/licenses/by/4.0/ This content is distributed under the terms of the Creative Commons Attribution 4.0 International license.

**FIG 4:**
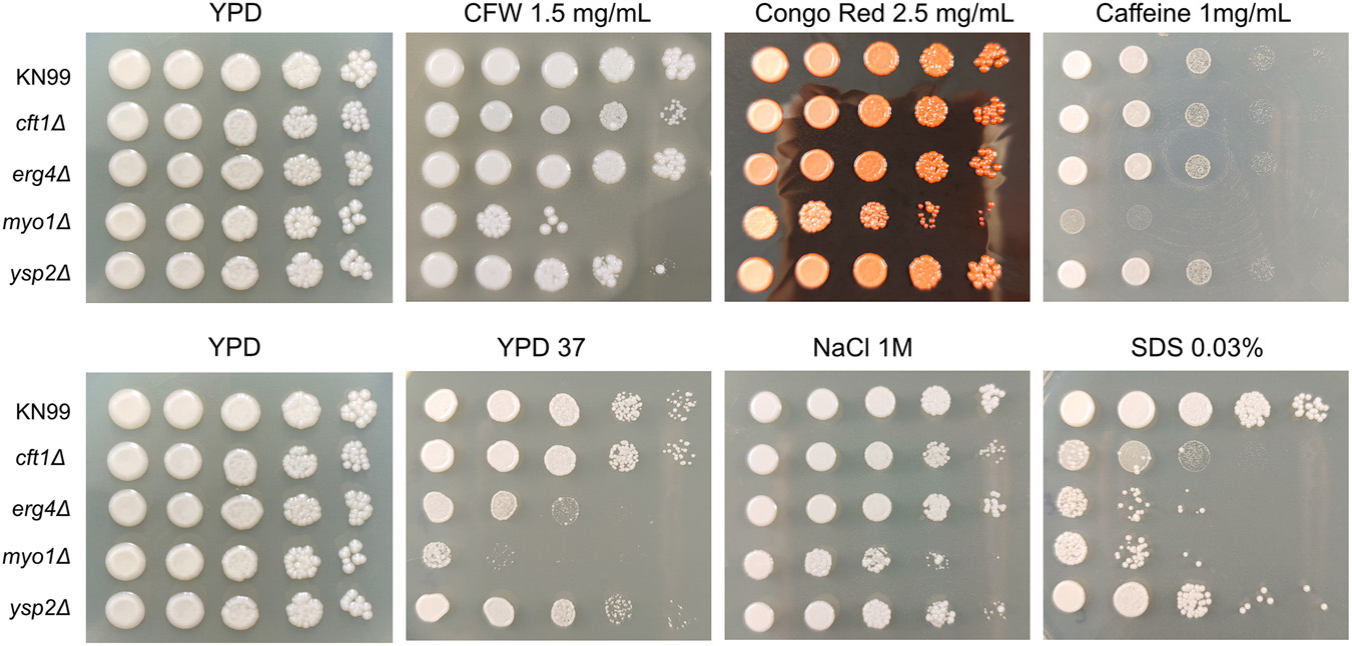
Caspofungin-sensitive deletion strains also showed sensitivity to membrane stress. Cell wall stress phenotypes of *cft1*Δ, *erg4*Δ, *myo1*Δ, and *ysp2*Δ strains were compared to those of the parental KN99 strain. Overnight cultures grown in YPD were diluted to an OD_650_ of 1 and serially diluted 1:10 four times before being spotted on agar plates containing the indicated stressor. The plates were incubated at 30°C for 3 days, except for a YPD plate whose stressor was growth at 37°C (YPD 37).

We examined the sensitivity to a variety of cell stressors. All four deletion mutants were significantly more sensitive than the WT to sodium dodecyl sulfate (SDS), supporting the possibility that their membranes might have been compromised (Fig. 4). The *myo1*Δ strain was the only strain to show significant sensitivity to the cell wall stressors calcofluor white (CFW), Congo red, and caffeine. The *erg4*Δ isolates also showed increased sensitivity to oxidative and nitrosative stress (Fig. S4), which may reflect a role for ergosterol in mitigating oxidative stress, as has been observed in Saccharomyces cerevisiae strains used in wine fermentation (18).

10.1128/msphere.00134-22.4 FIG S4 Sensitivities of the deletion strains and wild-type KN99 to oxidative and nitrosative stress. Overnight cultures grown in YPD were diluted to an OD_650_ of 1 and serially diluted 1:10 4 times. Plates were incubated at 30°C for 3 days. Download FIG S4, TIF file, 1.4 MB. Copyright © 2022 Moreira-Walsh et al. 2022 Moreira-Walsh et al. https://creativecommons.org/licenses/by/4.0/ This content is distributed under the terms of the Creative Commons Attribution 4.0 International license.

**FIG 5:**
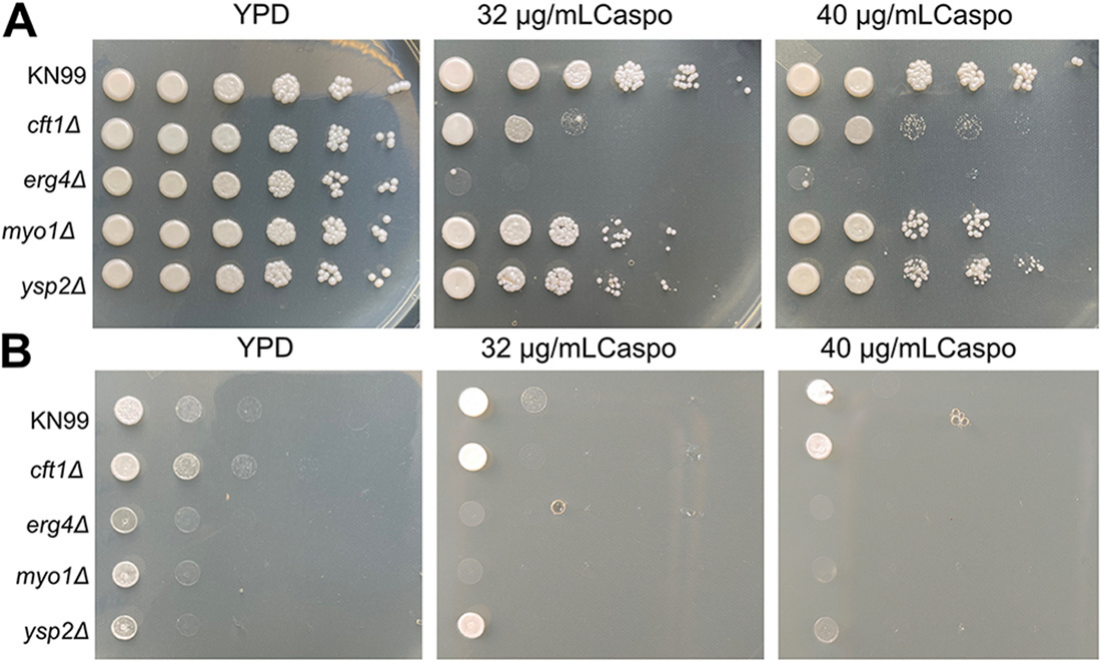
High temperature increases the sensitivity of deletion strains to caspofungin. The KN99 and *cft1*Δ, *erg4*Δ, *myo1*Δ, and *ysp2*Δ strains were grown overnight in YPD and diluted to an OD_650_ of 1 before being spotted in 4-step serial dilutions on YPD agar plates containing 32 or 40 μg/mL caspofungin. The plates were incubated for 3 days at 37°C (A) or 39°C (B).

Three deletion mutants, the *erg4*Δ, *myo1*Δ, and *ysp2*Δ strains, showed impaired growth at 39°C compared to the growth of KN99 (Fig. 4) and showed more sensitivity to caspofungin in a liquid assay than did the *cft1*Δ strain or the WT KN99. We found this lack of sensitivity of these three deletion mutants to other cell wall stressors surprising, given the importance of the caspofungin target protein, β-1,3-glucan synthase, to cell wall integrity. Thus, we wanted to explore whether the sensitivity of these deletion mutants to caspofungin was due to cell wall defects or because of alterations in the membrane structure or permeability. We first tested the sensitivity to caspofungin at higher temperatures and found that all four deletion mutants showed substantially increased sensitivity to caspofungin at 37°C (Fig. 5A) and all strains were profoundly or completely inhibited by caspofungin at 39°C, even the WT KN99 (Fig. 5B). This suggests that membrane fluidity can directly impact caspofungin sensitivity.

Since we found that the *erg4*Δ strain was sensitive to caspofungin and a previous screening also found that a deletion of *ERG3* increased caspofungin sensitivity (8), we explored whether the ergosterol content of membranes was altered in any of the deletion strains. Using a sterol quantification method developed for C. albicans (19), we confirmed that the *erg4*Δ cells had less ergosterol in their membranes but that the *erg4*Δ strain was the only one of the four deletion strains to show a significant reduction in ergosterol compared to the amount in KN99 (Fig. S5). Thus, we do not think that ergosterol content by itself can explain the resistance of cryptococcal cells to caspofungin.

10.1128/msphere.00134-22.5 FIG S5 Deletion of *ERG4* reduces ergosterol in the plasma membrane. KN99 and each deletion strain was grown overnight in 50 mL YPD. Membrane sterols were extracted using alcoholic potassium hydroxide and heptane. The presence of ergosterol and sterol intermediate 24(28) dihydroergosterol (DHE) was measured spectrophotometrically. The graph is representative of three independent replicates. Error bars represent the standard deviations in measurements of three technical replicates. Download FIG S5, EPS file, 0.1 MB. Copyright © 2022 Moreira-Walsh et al. 2022 Moreira-Walsh et al. https://creativecommons.org/licenses/by/4.0/ This content is distributed under the terms of the Creative Commons Attribution 4.0 International license.

### Chitin and chitosan levels and localization.

**FIG 6:**
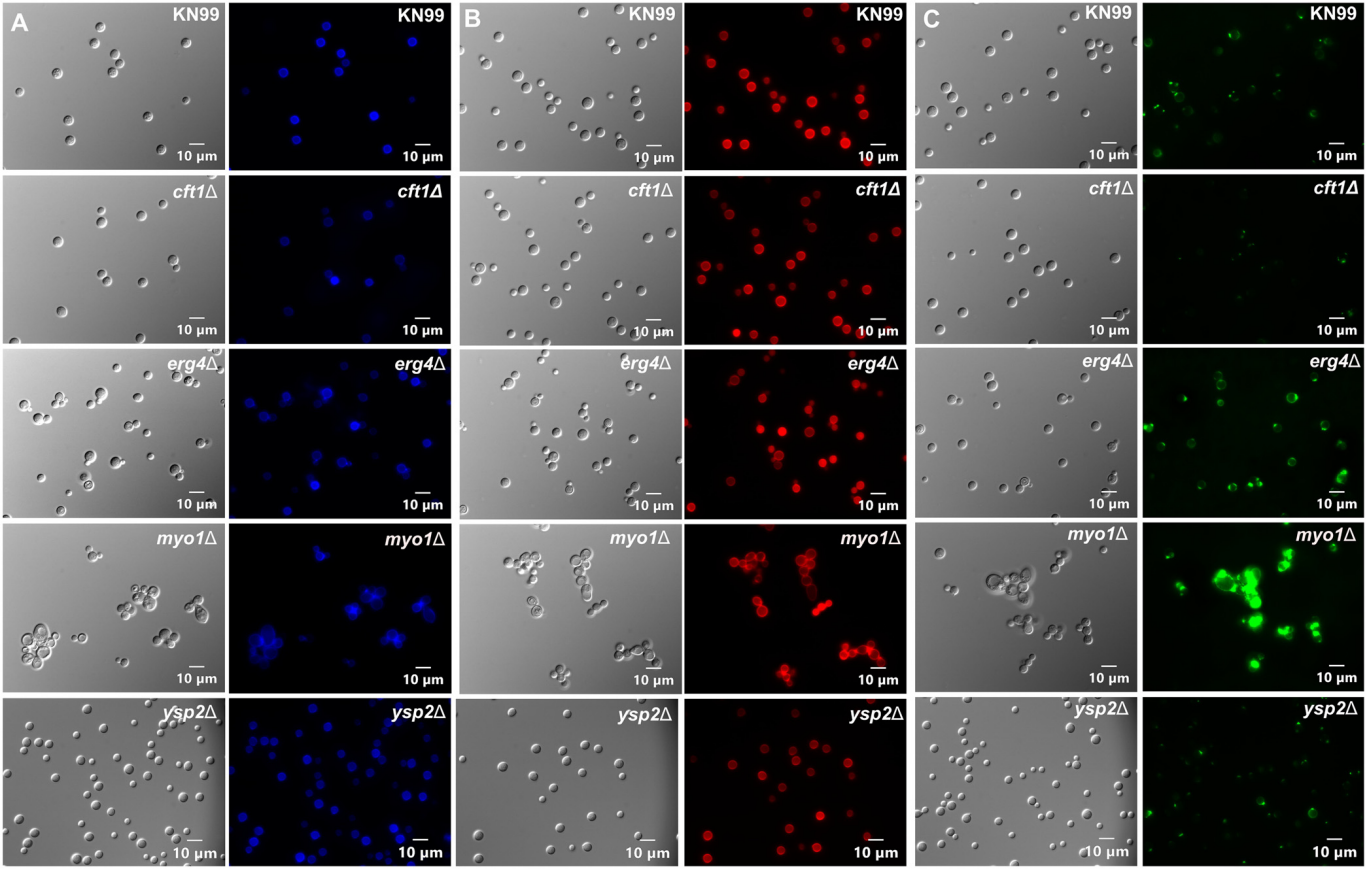
Deletion of *MYO1* altered yeast cell morphology, and deletions of either *ERG4* or *MYO1* increased WGA staining. Cell wall staining phenotypes of KN99 and the *cft1Δ*, *erg4Δ*, *myo1Δ*, and *ysp2Δ* deletion strains. Cells were incubated with calcofluor white (A), cibacron red (B), or wheat germ agglutinin-Alexa Fluor 488 (C). A differential interference contrast (DIC) image of the same slide section is shown to the left of each fluorescence image.

We next wanted to evaluate the amounts and localization of chitin and chitosan on the cell surface of these deletion mutants. We hypothesized that the increased sensitivity to caspofungin might be associated with mislocalization or altered levels of chitin and chitosan, as has been observed in a previous screen of deletion mutant strains with altered caspofungin sensitivity (7). We used CFW, which stains chitin, wheat germ agglutinin (WGA), which stains chitin oligomers, and cibacron red (CBR), which stains chitosan. Our approach of using fluorescent staining of the cell wall is based on the observation that cells lacking the calcineurin target and RNA-processing protein Puf4 also showed altered staining patterns with CFW and WGA (20). In this assay, the *myo1*Δ strain showed the most dramatic differences from KN99, with increased staining by all three dyes (Fig. 6). Visually, the *erg4*Δ strain had increased WGA staining and perhaps a modest decrease in CBR staining of the chitosan (Fig. 6B and C). We examined these *myo1*Δ and *erg4*Δ strains by confocal microscopy and observed increased WGA staining in both deletion strains compared to the WGA staining in KN99 (Fig. S6). This experiment was conducted prior to confirmation of the *ysp2*Δ strain. This suggests that the chitin may be more accessible, suggesting a more porous cell wall or altered morphology of the cell wall. However, we cannot rule out the possibility that WGA was binding to other biomolecules in the cell wall that were more accessible in these deletion strains.

10.1128/msphere.00134-22.6 FIG S6 Confocal imaging of KN99 (A), *erg4*Δ (B), and *myo1*Δ (C) cells using DIC (left) and stained with WGA (right). Download FIG S6, TIF file, 2.2 MB. Copyright © 2022 Moreira-Walsh et al. 2022 Moreira-Walsh et al. https://creativecommons.org/licenses/by/4.0/ This content is distributed under the terms of the Creative Commons Attribution 4.0 International license.

### Morphology of cell walls.

**FIG 7:**
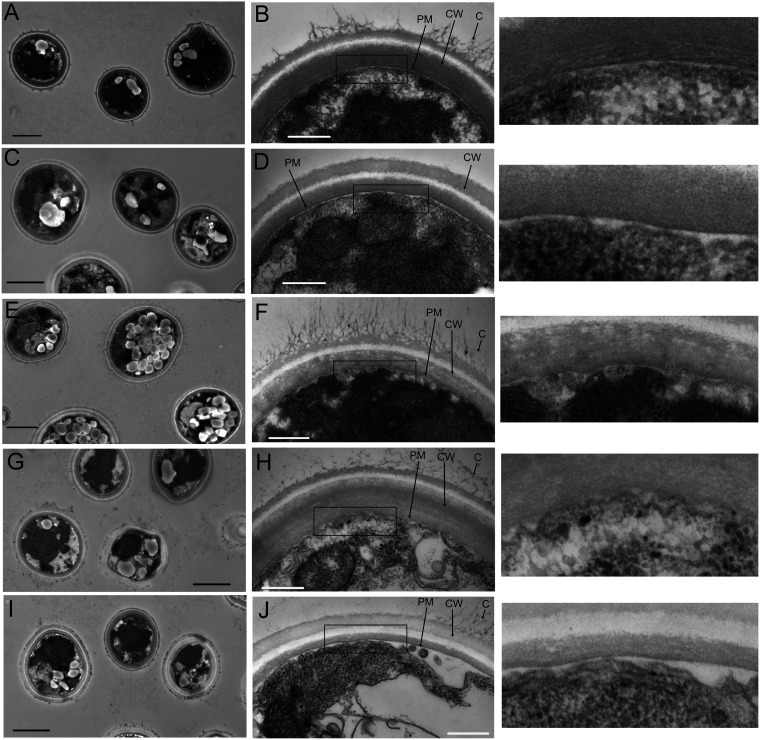
The cell walls of all deletion strain cells are intact. Transmission electron microscopy of KN99 cells at a magnification of ×3,000 (A), KN99 cells at ×25,000 (B), *cft1*Δ cells at ×3,000 (C), *cft1*Δ cells at ×25,000 (D), *erg4*Δ cells at ×3,000 (E), *erg4*Δ cells at ×25,000 (F), *myo1*Δ cells at ×3,000 (G), *myo1*Δ cells at ×25,000 (H), *ysp2*Δ cells at ×3,000 (I), and *ysp2*Δ cells at ×25,000 (J). The boxed areas in the ×25,000 magnification images are shown enlarged to the right of the original images. The scale bars show 2 μm at ×3,000 magnification and 500 nm at ×25,000 magnification. PM, plasma membrane; CW, cell wall; C, capsular material. Cells were grown overnight in YPD at 30°C prior to preparation for imaging. Sections were stained with uranyl acetate and lead acetate.

We wanted to examine the cell wall structure, and for this, we employed transmission electron microscopy (TEM) on cells fixed and stained with uranyl acetate and lead citrate. Representative images are shown in Fig. 7. At the lower magnification, KN99 cells showed a round cell with visible intact vesicles (Fig. 7A). At the higher magnification, the plasma membrane was a regular bilayer structure, and the wall appeared as three layers with some attached capsular material (Fig. 7B). The *cft1*Δ strain showed wild-type morphology at both magnifications, except for the lack of visible capsular material (Fig. 7C and D). The loss of *ERG4* caused a dramatic increase in lipid-containing vesicles visible at the lower magnification (Fig. 7E). At the higher magnification, the cell wall was intact, with the three visible layers and abundant attached capsule fibers. The plasma membrane showed two distinct layers but had a ruffled appearance (Fig. 7F and enlargement of boxed area to the right). The *myo1*Δ cells were less rounded, with an increased accumulation of lipid vesicles (Fig. 7G). At the higher magnification, the cell wall was intact, with three visible layers and attached capsule (Fig. 7H). The *myo1*Δ cells’ plasma membrane had two distinct layers but with a ruffled appearance similar to that of *erg4*Δ cells, as highlighted in the enlargement of the boxed area in Fig. 7H. The *ysp2*Δ cells were generally rounded, with some increase in lipid vesicles (Fig. 7I). The cell wall looked intact, with three layers, but had reduced capsule fibers and possibly thinner walls (Fig. 7J). The difference in staining intensities of the walls could be an artifact of the orientation of the cells relative to the section. Alternatively, it is known the uranyl/lead acetate stain preferentially stains protein and carbohydrates, so if the deletion mutants have altered accumulations of proteins or polysaccharides in their walls, it is possible that the staining intensity may be altered compared to that of KN99 cells.

### Membrane integrity.

We wanted to assess how membrane integrity affects caspofungin sensitivity directly. All four deletion strains showed increased susceptibility to the detergent SDS, so we asked whether caspofungin was synergistic with SDS in KN99 cells. We assessed this in a checkerboard assay and calculated a fractional inhibitory concentration index (FICI) of 0.25 for the combination of the detergent with caspofungin, indicating a synergistic interaction (Table S3) (21).

10.1128/msphere.00134-22.9 TABLE S2 List of primers used to create deletion strains. Download Table S2, DOCX file, 0.02 MB. Copyright © 2022 Moreira-Walsh et al. 2022 Moreira-Walsh et al. https://creativecommons.org/licenses/by/4.0/ This content is distributed under the terms of the Creative Commons Attribution 4.0 International license.

### Membrane visualization by fluorescent dyes.

**FIG 8:**
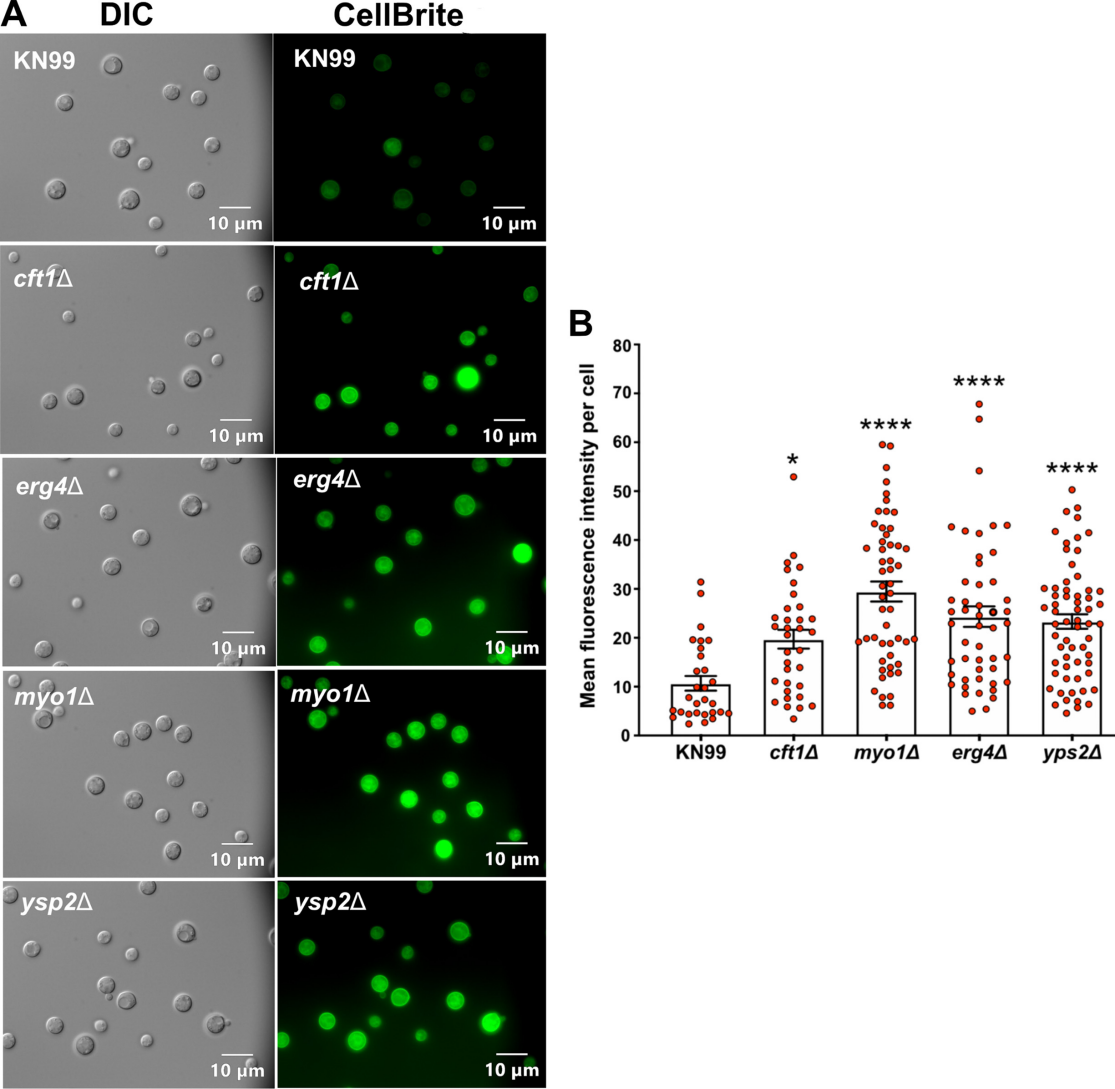
The membranes of the deletion strains were more exposed to membrane-specific dyes than the membranes of wild-type cells. Overnight cultures of KN99 and the four deletion strains were stained with CellBrite fix 488. The stained cells were directly imaged without fixing. (A) Representative DIC and fluorescent images of the five strains. (B) Mean fluorescence intensity was based on measuring the fluorescence of 30 or more cells per strain. The differences in mean fluorescence were evaluated using one-way ANOVA with Dunnett’s *post hoc* test. Error bars show standard deviations. *, *P* < 0.05; ****, *P* < 0.001.

To support our hypothesis that membrane alterations in these deletion strains result in greater caspofungin sensitivity, we attempted to visualize the membrane by fluorescence microscopy. We were unable to show any staining using DiOC_6_ (3,3′-dihexyloxacarbocyanine iodide), as has been done previously (10), but using the CellBrite fix dye, we showed that all four mutants had increased membrane staining compared to that of the WT (Fig. 8A). We measured the fluorescence of individual cells using ImageJ and compared the average fluorescence per cell between the strains using one-way analysis of variance (ANOVA) (Fig. 8B). All four deletion mutants had increased mean fluorescence, with the *erg4*Δ, *myo1*Δ, and *ysp2*Δ strains showing a greater difference from the WT (*P* < 0.001) than was observed between the *cft1*Δ strain and the WT (*P* < 0.05).

## DISCUSSION

### Comparison to other caspofungin screens.

The first screen of one of the Madhani deletion libraries for caspofungin sensitivity was conducted in YPD medium at 30°C (8). The authors reported that deletion of either *ERG3* or *ERG4* increased sensitivity to caspofungin at 8 μg/mL in liquid culture. We saw no difference in sensitivity for the *erg3*Δ strain in our assay, which may be due to the difference in the media used for the screens. More recently, Pianalto et al. screened the 2008 and 2015 Madhani deletion mutant libraries for caspofungin sensitivity (7, 14). They identified 14 deletion strains that met their criterion of being as sensitive to caspofungin or more so than the *cna1*Δ mutant strain. Interestingly, we observed no overlap with the deletion strains from the 2015 library that were identified as sensitive in our assay. Their screening medium was also YNB, but there were significant differences in the methodology that likely led to the identification of different strains as sensitive. The Pianalto assay used a significantly higher starting culture density, did not normalize for the growth of the different strains, and exposed the cells to caspofungin for 24 h versus 48 h in our assay (7). The cells for our assay were passaged two times for 48 h each time in YPD medium before being normalized by optical density (OD) and transferred to YNB medium, pH 7. Thus, the cells were likely older at the start of the screening assay than those used in the assay by Pianalto et al. (7). The age and density of the culture are known to impact the cell wall chitin and chitosan contents, as well as other virulence factors, and to increase the resistance of cells to macrophage uptake and host killing (22, 23). These results highlight the importance of using different methodologies to test for antifungal sensitivity.

### Impact of caspofungin on iron uptake.

*CFT1* encodes a high-affinity iron permease and has been characterized for its role in iron metabolism and contribution to various virulence factors (16). Deletion of this gene is known to reduce virulence in a mouse model of infection and increase sensitivity to both AMB and azole drugs. However, there are no published studies showing that deletion of *CFT1* affects resistance to caspofungin. Caspofungin may impact reductive iron uptake by its ability to bind to melanin and interfere with the reduction of Fe^3+^ to Fe^2+^ by melanin (24–26). This may explain the increased sensitivity of the *cft1*Δ strain to caspofungin but not to other cell wall-related stressors (Fig. 3 and 4). The loss of *CFT1* does significantly reduce iron uptake in a low-iron medium, and it has been shown to increase sensitivity to some azoles (16). We observed a slight increase in the sensitivity to AMB but no change in the sensitivity to FLC (Fig. S2). The previous assays for FLC and AMB MICs were done in YPD, and our experiments were done using YNB at pH 7. Perhaps the alterations to the membrane due to the higher pH, even in the *cft1*Δ background, were enough to change the antifungal response. We did not test caspofungin sensitivity in a low-iron medium, but the moderate increase in the sensitivity of the *cft1*Δ strain to caspofungin did not suggest that impaired iron transport played a major role in the resistance of C. neoformans to caspofungin.

### Caspofungin and ergosterol synthesis.

*ERG4* encodes a C-24(28) sterol reductase and is the closest homolog to *ERG4* in S. cerevisiae, which catalyzes the final step in ergosterol biosynthesis to synthesize ergosterol from 5,7,22,24(28) ergostatetraenol (27). It was previously identified as contributing to caspofungin sensitivity in a screen using a different medium and growth conditions and has been shown to play a role in the response to higher pH (8, 28). The dramatically increased sensitivity of the *erg4*Δ strain to caspofungin may be due to a combination of factors. The altered membrane composition appeared to alter the deposition or exposure of the cell wall chitin, based on the observed increase in WGA staining (Fig. 6, Fig. S5). An altered cell wall could disrupt or alter the orientation of the β-1,3-glucan synthase, rendering it more accessible to inhibition by caspofungin, although the cell wall appeared to be intact by TEM analysis (Fig. 7E and F). The altered membrane morphology and increased lipid accumulation suggested significant defects in the plasma membrane. The much lower MIC for FLC in the *erg4*Δ strain suggested that the combination of the *ERG4* deletion with inhibition of *ERG11* rendered the cells much more susceptible to azoles. The two independent *erg4*Δ isolates appeared to be slightly more resistant to AMB than WT cells. Recent studies of the membrane contents in AMB-resistant Candida haemulonii strains revealed that most of the membrane sterols are composed of ergosterol pathway intermediates that are more resistant to binding by AMB (29).

### Caspofungin and myosin.

The *MYO1* gene encodes a heavy chain of myosin but has not yet been characterized in C. neoformans. Its protein sequence is most like that of Myo1p in S. cerevisiae, and mutants with deletions of *MYO1* in S. cerevisiae showed altered chitin deposition, loss of cell type-specific budding patterns, and defective cell wall synthesis at the mother-bud neck (30). The cell wall stress phenotypes observed here support the hypothesis that deletion of *MYO1* disrupts cell wall synthesis or homeostasis. The deletion strain was slower growing, which might reflect a defect in cytokinesis that could result from the loss of the myosin heavy chain. The *myo1*Δ strain was also quite sensitive to caspofungin and showed the most deformed cellular morphology by microscopy, with cell separation defects and enlarged cells (Fig. 6). Not surprisingly, *myo1*Δ cells were sensitive to a variety of stressors, grew more slowly, and showed more cell wall staining, suggesting that the cell wall was also defective. Surprisingly, the cell walls did not show substantial defects by ultrastructural analysis (Fig. 7). We hypothesize that the deletion of *MYO1* may not affect caspofungin resistance specifically, but is more likely to disrupt vesicle transport, which would impair synthesis and remodeling of the plasma membrane, perhaps altering the orientation or accessibility of the caspofungin target β-1,3-glucan synthetase. Myo1p also contains an E3 ubiquitin ligase ring domain, so a role for protein turnover in resistance to caspofungin cannot be ruled out.

### Caspofungin and sterol transport.

The *YSP2* gene encodes a protein containing VASt, GRAM, and Pleckstrin homology domains that has not been characterized in C. neoformans. In S. cerevisiae, the homolog is localized to the endoplasmic reticulum (ER) and most likely has a role in sterol transport (17, 31). In S. cerevisiae, deletion of *YSP2* results in AMB sensitivity (17). Our deletion strain shows significant increases in sensitivity to FLC and AMB (Fig. S2), supporting the hypothesis that the sterol content of the membrane may be altered in this deletion strain. However, we did not observe a decrease or increase in ergosterol content relative to that of WT KN99 (Fig. S4). The mechanism by which disruption in sterol transport affects caspofungin sensitivity is not known, but alterations in sterol distribution affect the membrane fluidity and may allow greater influx of caspofungin into the cells. Alternatively, membrane changes may alter the localization of Fks1p, rendering it more sensitive to inhibition by caspofungin. Recently, a research group deleted multiple ergosterol biosynthetic genes that function late in the pathway. They found that deletion of *ERG6* rendered the cells sensitive to micafungin, albeit at very high concentrations (32). This supports the hypothesis that the overall sterol composition can change the sensitivity to cell wall inhibitors.

In summary, we conclude that the membranes of the deletion mutant strains are substantially altered and possibly more permeable than those of WT cells. These alterations may allow caspofungin to accumulate at a high concentration intracellularly, or the orientation of Fks1p may be more accessible in these deletion strains. These data, in the context of other studies that have identified deletions that result in increased caspofungin sensitivity, support the hypothesis that Cryptococcus has multiple strategies by which it can resist inhibition by the echinocandins. Going forward, the most likely path to new therapies will be the combination of caspofungin with inhibitors that can substantially alter the composition or morphology of the cryptococcal cell wall or membrane.

## MATERIALS AND METHODS

### Strains, media, and chemicals.

KN99α, a strain of C. neoformans serotype A (33), was used as the wild-type strain. The 2015 and 2016 gene deletion strain libraries were obtained as frozen cultures in 96-well plates from the Fungal Genetics Stock Center (14).

Strains were grown on YPD (1% yeast extract, 2% Bacto peptone, and 2% dextrose). Solid medium contained 2% Bacto agar. Selective YPD medium contained 100 μg/mL nourseothricin (NAT) (GoldBio, USA) and/or 200 μg/mL G418 (geneticin; Gibco Life Technologies, USA). YNB 2% (0.67% yeast nitrogen base, 2% dextrose, pH 7.0, with 50 mM MOPS [morpholinepropanesulfonic acid]) was used for all screening and limiting dilution inhibition assays unless otherwise noted. The medium was prepared in deionized water passed through the ion exchange Milli-Q (MQ) filter system (Sigma-Millipore, USA).

### Screening for caspofungin sensitivity.

The method of screening for caspofungin sensitivity is briefly described here. Additional details are available in the supplemental material (Text S1). The schematic of the assay is depicted in Fig. 1. Each well of the frozen 96-well plates from the library stock plates was pin replicated onto YPD agar, and the replicates allowed to recover for 48 h at 25°C (Fig. 1a and b). We passaged each plate twice in liquid YPD to allow the cells to fully recover and reach sufficient cell density for the normalization step (Fig. 1c and d). We found that normalizing each strain within a plate to the same optical density was essential to ensuring reproducible results (Figure 1e and f). After normalization, all deletion strains were diluted in series to an optical density at 650 nm (OD_650_) of 0.001 before adding caspofungin to 3 technical replicates, with 1 replicate plate left untreated as a control (Fig. 1g to j). All incubation steps in liquid medium (Fig. 1b to j) were carried out in plate shakers at 25°C. We employed a metabolic readout based on cellular dehydrogenase and/or reductase enzymatic reduction of a tetrazolium salt to an orange formazan compound that is readily quantified using a plate reader (Fig. 1k and l) (34, 35). This reduced the inherent variability of an optical density assay, particularly with deletion strains, whose cells tend to clump. The data were analyzed in Prism, and the output calculated as the percentage of that of the untreated replicate averaged across the three treated replicates. Sensitive strains were defined as showing 50% or more inhibition of XTT activity relative to the mean of the normalized output across the entire plate.

### Generation of gene deletion strains.

All deletion primers used in this study are listed in Table S2. The deletion strains were produced by generating deletion cassettes where the targeted gene was replaced with a geneticin (G418)-selectable marker using a double-joint PCR (DJ-PCR), as described previously (36, 37). The constructs were transformed into C. neoformans KN99α by biolistic transformation (38–40). Transformants were screened on YPD medium supplemented with 150 μg/mL geneticin and confirmed by PCR screening to certify the insertion of the allele.

10.1128/msphere.00134-22.8 TABLE S1 List of the 17 deletions strains from the 2015 and 2016 C. neoformans deletion library that showed a significantly increased sensitivity to caspofungin in a dose dependence assay. Download Table S1, DOCX file, 0.02 MB. Copyright © 2022 Moreira-Walsh et al. 2022 Moreira-Walsh et al. https://creativecommons.org/licenses/by/4.0/ This content is distributed under the terms of the Creative Commons Attribution 4.0 International license.

### Agar plate phenotyping.

Overnight cultures were washed with phosphate-buffered saline (PBS) and resuspended in PBS to an OD_600_ of 1. Cells were 10-fold serially diluted, and 2.5 μL of each dilution was spotted onto YPD agar plates alone at 39°C, to induce heat stress, and YPD agar plates alone and stress plates at 30°C. Each stress plate was composed of YPD medium supplemented with the indicated concentrations of the following compounds: 0.3% sodium dodecyl sulfate (SDS; Sigma) and 32 μg/mL or 40 μg/mL caspofungin acetate (Biosynth Carbosynth), to investigate cell integrity stress; 2 mM tert-butyl hydroperoxide (tBOOH; Spectrum Chemical) and 0.75 mM NaNO_2_ (Sigma), to impose nitrosative and oxidative stress; and 0.5 mg/mL calcofluor white (CFW; MP Biomedicals), 1 mg/mL caffeine (Sigma), and 2.5 mg/mL Congo red (Santa Cruz Biotechnology) to induce cell wall stress. Plates were incubated at 30°C, unless otherwise noted, and photographed after 3 and 5 days of incubation.

### Determination of MIC.

Assays to determine the MICs needed to inhibit C. neoformans growth relative to that of vehicle-treated cells were done as described previously with slight modifications (41). Cells were grown overnight in 4-mL cultures of YPD at 30°C with shaking and then diluted to an OD_650_ of 0.001 in YNB 2% or YNB 2% plus 1% dimethyl sulfoxide (DMSO), depending on the vehicle used for dissolving the inhibitor. The treated and control cells were incubated in clear, round-bottom 96-well plates covered in Breathe-Easy membrane without shaking for 48 h at 30°C or 35°C, and the optical density measured. MICs were determined using concentrations of compounds over 5-step dilution ranges of 2 to 32 μg/mL for caspofungin, 1.56 to 25 μM (0.48 to 7.6 μg/mL) for fluconazole, and 0.39 to 6.25 μM (0.36 to 5.7 μg/mL) for amphotericin B. Each assay was done in triplicate, and all reported values are the averages of two or more independent assays. The data are presented as the average cell density as the percentage of that of untreated cells.

### Fluorescence microscopy.

Cells were picked up from a fresh agar plate using a 5-mL loop and transferred to 5 mL YNB, pH 7. Cultures were incubated for 16 to 18 h at 30°C with shaking at 300 rpm. Overnight cultures were collected by centrifugation at 3,500 × *g* for 8 min at 4°C and resuspended in 1× PBS at an OD_650_ of 1. For staining with calcofluor white (CFW), Alexa Fluor 488-conjugated wheat germ agglutinin (WGA; Invitrogen W11261), and fluorescein isothiocyanate (FITC)-conjugated concanavalin A (ConA; Sigma C7642), cells were fixed with 4% paraformaldehyde for 30 min on ice. Fixed cells were washed two times with PBS and were blocked with 2% bovine serum albumin (BSA) (product number A7906; Sigma) at 30°C for 30 min in a nutating mixer. Live cells were stained using the CellBrite fix 488 kit (catalog number 30090-T; Biotium). CFW, WGA, and ConA were used at 5 μg/mL, 100 μg/mL, and 50 μg/mL respectively. CellBrite stock solution was prepared at 1,000×, and 1 μL of the stock added to 100 μL cell suspension. Cells were incubated with the stains for 30 min at 4°C in the dark. Stained cells were washed with PBS three times and were spotted onto the slides. Slides were imaged using an Olympus BX61 with a 63× objective. For imaging, WGA was detected using the green fluorescent protein (GFP) settings and CFW was detected using the DAPI (4′,6-diamidino-2-phenylindole) settings in a Zeiss Axio Imager M2 equipped with a Hamamatsu Flash 4.0 (CMOS) camera.

### Transmission electron microscopy.

Wild-type KN99 and the deletion strains were grown in YNB medium buffered to pH 7 with MOPS for 48 h at 30°C. Cells were fixed in 2% paraformaldehyde/2.5% glutaraldehyde (Polysciences, Inc., Warrington, PA) in 100 mM sodium cacodylate buffer, pH 7.2, for 2 h at room temperature and then overnight at 4°C. Samples were washed in sodium cacodylate buffer and postfixed in 1% osmium tetroxide (Polysciences, Inc.) for 1 h at room temperature. Samples were then rinsed extensively in distilled water (dH_2_0) prior to *en bloc* staining with 1% aqueous uranyl acetate (Ted Pella, Inc., Redding, CA) for 1 h. Following several rinses in dH_2_0, samples were dehydrated in a graded ethanol series and embedded in Eponate 12 resin (Ted Pella, Inc.). Sections of 95 nm were cut with a Leica ultracut 7 (UC7) ultramicrotome (Leica Microsystems, Inc., Bannockburn, IL), stained with uranyl acetate and lead citrate, and viewed on a JEOL 1200 EX transmission electron microscope (JEOL USA, Inc., Peabody, MA) equipped with an AMT 8-megapixel digital camera and AMT Image Capture Engine V602 software (Advanced Microscopy Techniques, Woburn, MA).

### Sterol quantification method.

Extraction of total intracellular sterols was performed as previously reported by Arthington-Skaggs et al. (19) with a few modifications. All strains were grown overnight in 50 mL of YPD liquid medium at 30°C with shaking. Cells were pelleted by centrifugation at 2,500 rpm for 8 min and washed with sterile MQ water. The cell pellet was weighed and resuspended in 3 mL of 25% alcoholic potassium hydroxide. The resuspended cells were transferred to 16- by 100-mm sterile borosilicate glass screw-cap tubes and incubated for 2 h in an 85°C water bath. After incubation, the tubes were cooled to room temperature. The sterol extraction steps were carried out by the addition of 1 mL sterile MQ water and 3 mL *n*-heptane, followed by vortexing the tubes for 3 min. The tubes were allowed to stand for 30 min, and once clarified, the *n*-heptane layer was transferred to sterile borosilicate glass screw-cap tubes and stored overnight at −20°C. Samples were diluted 5-fold in 100% ethanol and scanned spectrophotometrically between 230 and 282 nm. The ergosterol content was calculated using the following equations: % ergosterol + % 24(28)DHE = [(*A*_281.5_/290) × *F*]/pellet weight, % 24(28)DHE = [(*A*_230_/518) × *F*]/pellet weight, and % ergosterol = [% ergosterol + % 24(28)DHE] − % 24(28)DH, where *F* is the factor for dilution in ethanol and the extinction coefficient values were previously determined as 290 for crystalline ergosterol and 518 for 24(28) dihydroergosterol (DHE).

### Checkerboard assay.

To evaluate the impact of SDS in combination with caspofungin in the deletion strains, a checkerboard assay was conducted in YNB 2%, pH 7. A broth microdilution method was performed in a 96-well round-bottom plate, using rows A to F and columns 1 to 10 for titrations between SDS and caspofungin. Row H was used as a control for SDS only, column 2 for a caspofungin control, and column 1 for a cell growth control. The concentrations ranged from 0.03% to 0.0002% for SDS and from 64 μg/mL to 2 μg/mL for caspofungin. Cells were grown overnight and diluted to an OD_650_ of 0.001 in YNB 2%. Plates were incubated at 30°C, and the OD_650_ was measured after 48 h. The fractional inhibitory concentration index (FICI) model is expressed as ΣFIC = FIC*_A_* + FIC*_B_* = MIC*_A_*_′_/MIC*_A_* + MIC*_B_*_′_/MIC*_B_*, where MIC*_A_* and MIC*_B_* are the MICs of agents *A* and *B* used alone and MIC*_A_*_′_ and MIC*_B_*_′_ are the MICs of agents *A* and *B* used in combination. The interaction between two compounds is considered synergistic when the FICI is ≤0.5, indifferent when the FICI is between >0.5 and 4, and antagonistic when the FICI is >4 (21, 42).

10.1128/msphere.00134-22.7 TEXT S1 Detailed description of the caspofungin screening assay of Cryptococcus neoformans. Download Text S1, DOCX file, 0.02 MB. Copyright © 2022 Moreira-Walsh et al. 2022 Moreira-Walsh et al. https://creativecommons.org/licenses/by/4.0/ This content is distributed under the terms of the Creative Commons Attribution 4.0 International license.

10.1128/msphere.00134-22.10 TABLE S3 MICs for SDS and caspofungin in checkerboard assay with wild-type KN99 strain. Assay was incubated for 2 days at 30°C. Download Table S3, DOCX file, 0.01 MB. Copyright © 2022 Moreira-Walsh et al. 2022 Moreira-Walsh et al. https://creativecommons.org/licenses/by/4.0/ This content is distributed under the terms of the Creative Commons Attribution 4.0 International license.
