# Supplementary figures and images for: Membrane Integrity Contributes to Resistance of Cryptococcus neoformans to the Cell Wall Inhibitor Caspofungin
Source: mSphere. 2022 Jun 27;7(4):e00134-22. doi: 10.1128/msphere.00134-22 (PMC9429927; doi:10.1128/msphere.00134-22)

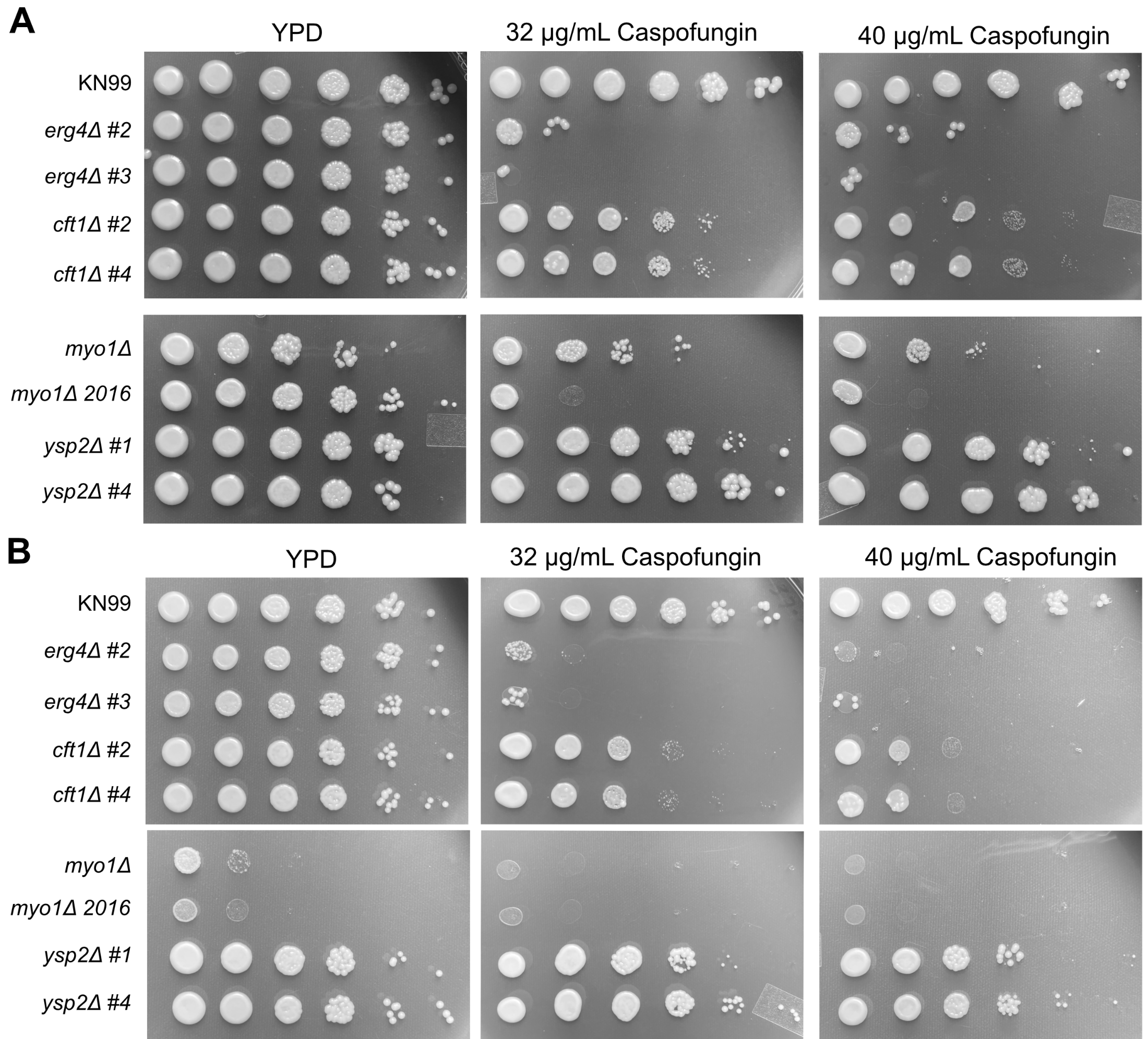

Supplement: FIG S2 [file msphere.00134-22-s0005.tif]

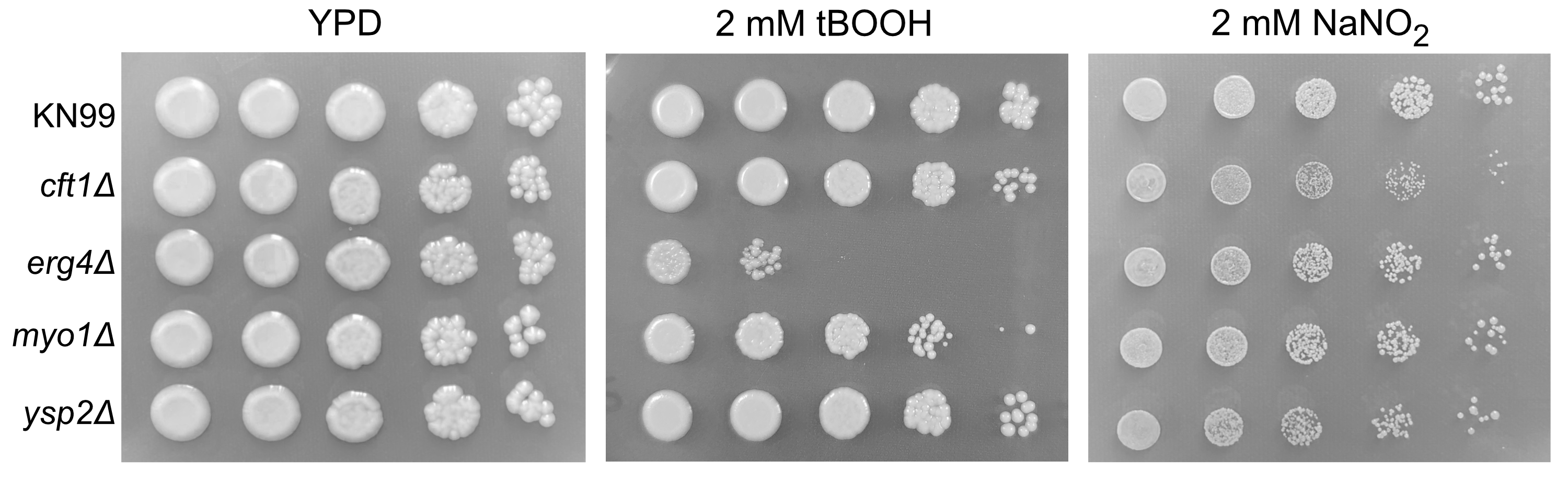

Supplement: FIG S4 [file msphere.00134-22-s0007.tif]

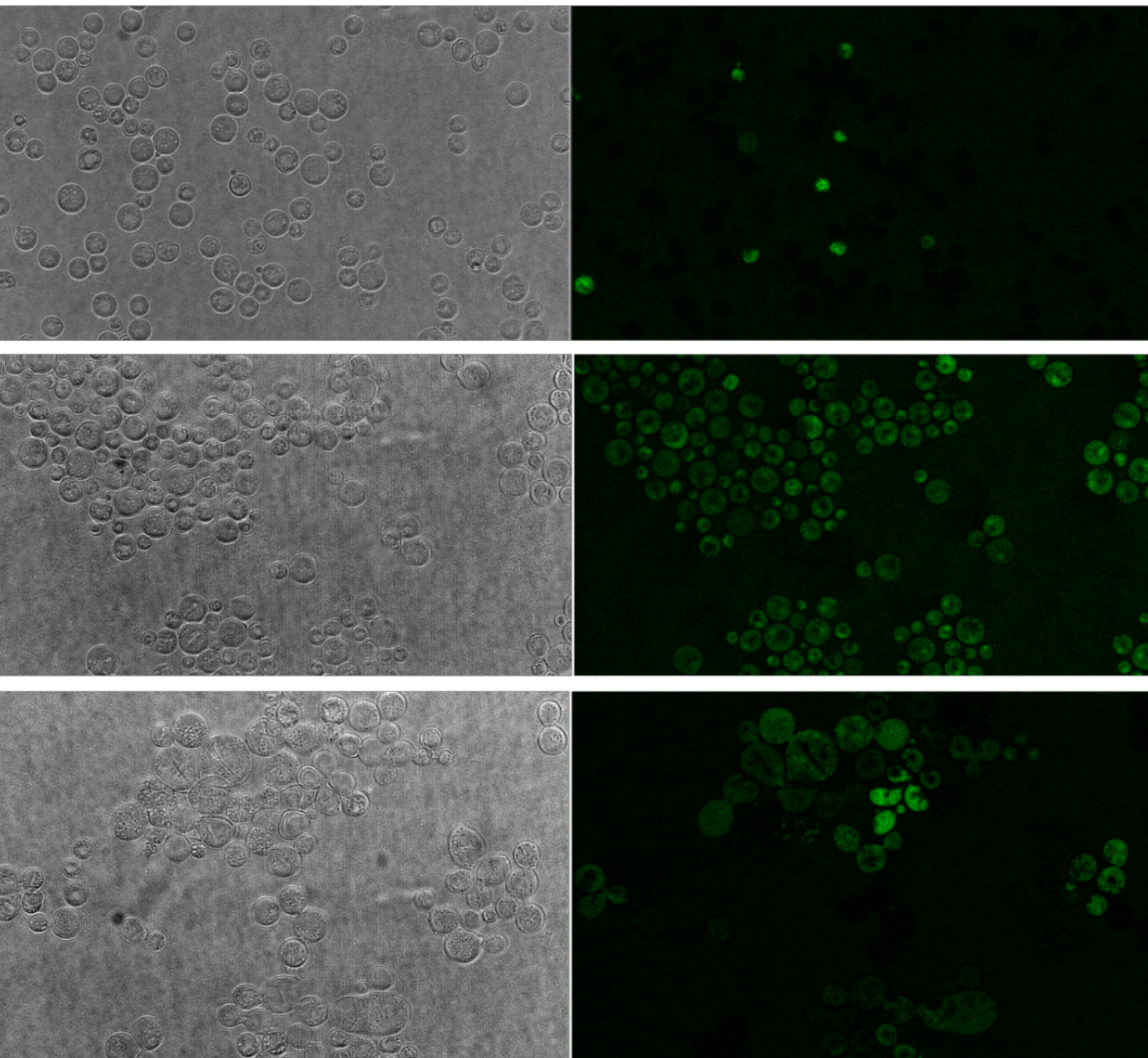

Supplement: FIG S6 [file msphere.00134-22-s0009.tif]
