# Supplementary material for: Membrane Integrity Contributes to Resistance of Cryptococcus neoformans to the Cell Wall Inhibitor Caspofungin
Source: mSphere. 2022 Jun 27;7(4):e00134-22. doi: 10.1128/msphere.00134-22 (PMC9429927; doi:10.1128/msphere.00134-22)
Supplement: TABLE S2 [file msphere.00134-22-s0002.docx]

**Supplemental Table 2:** Primers used to create gene deletions.

| **Name** | **Targeted gene** | **Sequence 5’-3’** |
| --- | --- | --- |
| **Deletion primers** | | |
| P1_Erg4 | Erg4 | GCAAGCCATTCTGGACACTG |
| P2_Erg4 | Erg4 | GATTGTGGGCGGGTAGAAAG |
| P3_Erg4 | Erg4 | GCCCACTGCAAAACATAAATAcaggctgcgaggatgtga |
| P4_Erg4 | Erg4 | tcacatcctcgcagcctgTATTTATGTTTTGCAGTGGGC |
| P5_Erg4 | Erg4 | cactggccgtcgttttacaacCATGATTTGACGGCGTGTTA |
| P6_Erg4 | Erg4 | TAACACGCCGTCAAATCATGgttgtaaaacgacggccagtg |
| P7_Erg4 | Erg4 | GTTGCTGGCATGACTCTTGA |
| P8_Erg4 | Erg4 | TACCCGCACAGGGTCCTTATA |
| P9_Erg4 | Erg4 | TGAAACCCATGAGAGCGATTAAG |
| P10_Erg4 | Erg4 | GTTGCCTTGTTCTCCACTCTTATG |
| P1_Myo1 | Myo1 | GGTGTACCGACGAGAATGTG |
| P2_Myo1 | Myo1 | AATCTTCCAGGCCCATATGATG |
| P3_Myo1 | Myo1 | TATACACCCCACGCAACTCAcaggaaacagctatgaccatg |
| P4_Myo1 | Myo1 | catggtcatagctgtttcctgTGAGTTGCGTGGGGTGTATA |
| P5_Myo1 | Myo1 | cactggccgtcgttttacaacAACAATGATGGGGAAAGTAGGA |
| P6_Myo1 | Myo1 | TCCTACTTTCCCCATCATTGTTgttgtaaaacgacggccagtg |
| P7_Myo1 | Myo1 | GGAAGTCGTGGAAAACTCGAT |
| P8_Myo1 | Myo1 | GCAAGGATCATCTAGGGGCTAC |
| P9_Myo1 | Myo1 | TGGTTGAAGATGGTGGCGGTTC |
| P10_Myo1 | Myo1 | GAGGTTGCTGAACATCGGAAC |
| P1_Cft1 | Cft1 | GCCGTGAGAATGAAGGGATG |
| P2_Cft1 | Cft1 | GTATGCAGTGCAAGTGACTGGT |
| P3_Cft1 | Cft1 | ATGCCCATAGTCCAGGTTTTCTCcaggaaacagctatgaccatg |
| P4_Cft1 | Cft1 | catggtcatagctgtttcctgGAGAAAACCTGGACTATGGGCAT |
| P5_Cft1 | Cft1 | cactggccgtcgttttacaacGGGGAGGCATTGAGGTTATTC |
| P6_Cft1 | Cft1 | GAATAACCTCAATGCCTCCCCgttgtaaaacgacggccagtg |
| 7_Cft1 | Cft1 | CCGCCCAGAACACTGTACAAC |
| P8_Cft1 | Cft1 | AACATCCTACATCCCCTCCC |
| P9_Cft1 | Cft1 | CGAGTGAACCATGGAGCATAA |
| P10_Cft1 | Cft1 | CCATTTTCGGCTGGAGTAAGTT |
| P1_Ysp2p | Ysp2 | GTGACGCCCATCTACAAAGAA |
| P2_ Ysp2p | Ysp2 | GAGGTTGGGAAACAAGGAAAG |
| P3_ Ysp2p | Ysp2 | CCGGCAGTCTCTTCATCGAAAcaggctgcgaggatgtga |
| P4_ Ysp2p | Ysp2 | tcacatcctcgcagcctgTTTCGATGAAGAGACTGCCGG |
| P5_ Ysp2p | Ysp2 | cactggccgtcgttttacaacCTCGTACCAACTCTGGCTCTG |
| P6_ Ysp2p | Ysp2 | CAGAGCCAGAGTTGGTACGAGgttgtaaaacgacggccagtg |
| P7_ Ysp2p | Ysp2 | AGGGAATAGGGAGAGGAGGAG |
| P8_ Ysp2p | Ysp2 | ACTTGGCAGAACTTTCTGGGT |
| P9_ Ysp2p | Ysp2 | AACTAAGATGGGAATGGGGTG |
| P10_ Ysp2p | Ysp2 | CATGTCGGCACTTTCTCTCTC |
| **PCR screening primers** | | |
| Primer A |  | TCTCCTCCGACAACCATACACTCAGC |
| Primer B |  | AGTTTGGTCGCTCTCTGTACC |
| **Gene specific primers** | | |
| F_GenSp_Erg4 | Erg4 | GGTCTATCCTTAATCGCTCTC |
| R_GenSp_Erg4 | Erg4 | CCAGAAGATCAACATCCATCC |
| F_GenSp_Myo1 | Myo1 | GAGAGGTTAGAAGCTAGTGGA |
| R_GenSp_ Myo1 | Myo1 | ACGAAGTGACTATCAGCGTA |
| F_GenSp_Cft1 | Cft1 | CCAACGAACTCTATTACCGAC |
| R_GenSp_ Cft1 | Cft1 | TGAGATAACGACTGACCTCTG |
| F_GenSp_Ysp2 | Ysp2 | CACCCCATTCCCATCTTAGTT |
| R_GenSp_Ysp2 | Ysp2 | GAGAGAGAAAGTGCCGACATG |

**Supplemental Table 3:** SDS and caspofungin assay for synergy

| **Strain** | **MIC_50_** | | | | **FICI** |
| --- | --- | --- | --- | --- | --- |
|  | **Alone** | | **Combined** | |  |
|  | **SDS** | **Caspofungin** | **SDS** | **Caspofungin** |  |
| KN99 | 0.015% | 32 ug/mL | 0.009% | 8 ug/mL | 0.25 |
