# Supplementary material for: Membrane Integrity Contributes to Resistance of Cryptococcus neoformans to the Cell Wall Inhibitor Caspofungin
Source: mSphere. 2022 Jun 27;7(4):e00134-22. doi: 10.1128/msphere.00134-22 (PMC9429927; doi:10.1128/msphere.00134-22)
Supplement: TEXT S1 [file msphere.00134-22-s0010.docx]

**Supplementary methods**

**Description:** using XTT Assay to measure the metabolic activity of *C. neoformans* deletion strains in response to treatment with caspofungin

**Reagents:**

-(A) Caspofungin diacetate [Sigma SML0425-5M; Carbosynth, FC16269]

-(B) XTT sodium salt: [Sigma: X4251 discontinued; GoldBio: X200]

-(C) Menadione sodium disulfate: [Sigma M2518-100G]

-YNB pH 7.0 (Difco: yeast nitrogen base without amino acids2% glucose(Difco

-YPD, yeast extract, peptone, dextrose

-PBS, phosphate buffered saline, pH 7

*Stock solutions:*

Caspofungin at 5mg/mL in milliq H_2_O

XTT at 0.5g XTT sodium salt per 1L of 1X sterile PBS; store at -80°C in 10 ml aliquots

Menadione at 10mM in 100% acetone; store at -80°C in 15 μl aliquots

*C. neoformans* deletion libraries (from Fungal Genetic stock center)

UCSF (Madhani) 2015 deletion library

UCSF (Madhani) 2016 deletion library

**Equipment and disposables:**

(1) 96 well Deep well plate [Thermo Scientific: 27874], plastic, opaque, max capacity of 2L

(2) Costar Assay Plate, 96-well clear round bottom with lid [Fisher: 07-200-760 (3788)]

(3) Costar Assay Plate, 96-well clear flat bottom with lid [Fisher: 07-200-656 (3370)]

(4) Breathe Easy Sealing Membrane 100/pk [RPI research Products Int.: BEm-1]

(5) Shaker; Digital, Microplate, 11" x 10.6", Thermo Fisher, 120V

(6) Microplate absorbance spectrophotometer

(7) Large benchtop refrigerated centrifuge equipped with a rotor that can hold 96 well plates

**Retrieval of Cells from Freezer stocks:**

1. The UCSF deletion library strains are stored in 96-well flat bottom microtiter plates. Cells are suspended in 200 μL YPD+30% glycerin media and covered with a protective sealing membrane.
2. Cells are transferred to liquid working stocks in two ways:
   1. Solid: a 96-pronged replicator is used to take up frozen cells from 96-well stock plate (3) and deposit them onto YPD-agar plate, which is incubated at 25%°C. Grown cells are then collected and added to 600 μL YPD in a deep well (1) and plates are shaken at 650 rpm (5) for 48 hours at room temperature
   2. Liquid: cells are allowed to defrost in the 96-well stock plate (3), and 30 μL of liquid cells are added to 600 μL YPD in a deep well plate (1). Plates are shaken at 650 rpm for 48 hours at room temperature (5).
   3. the resulting ‘working stock’ is then used for the next steps

**Growth, treatment and assay methods:**

I. Preparation of Cells

1. In a deep well deep plate (1) add 30 μL cell culture to 600 μL YPD. [note this is the second passage in liquid]
2. Incubate plates at room temperature for 2 days with shaking at 650 rpm (5)
3. Wash cells in PBS 2 times, decanting liquid each time
4. Add 200 μL PBS and pipette up and down to completely re-suspend cells
5. Using flat bottom 96 well plate (3) measure OD_650_ in a plate reader (6); convert OD to volume needed to normalize to OD of 2 in 120 μL [refer to excel file “Plate normalization template”]
6. Set up round bottom 96-well plate (2) with 120 μL PBS, and displace cell culture amounts, keeping final well volumes at 120 μL (discard PBS and add same volume of cell culture)

II-A. Cell Dilutions and Caspofungin Treatment (deletion library)

1. *Dilution 1*: in round bottom plate (2) make initial dilution: add 10 μL normalized cell culture to 190 μL YNB pH 7.0 (OD=2.0 to OD=0.1)
2. *Dilution 2:* Use YNB pH 7.0 to dilute cells a second time to 1:10, so that wells are equal to 100 μL, in round bottom 96-well plates (2) (10 μL of 1 OD cell culture + 90 μL YNB pH 7.0; OD=0.1 to OD=0.01)
3. *Dilution 3:* Use YNB pH 7.0 to dilute cells a third time (10 μL of 1 OD cell culture + 80 μL YNB pH 7.0; OD=0.01 to OD=0.001) in 4 replicate plates
4. *Add caspofungin:* dilute 5 mg/mL caspofungin (A in sterile YNB pH 7 to 100 μg/ul and then add 10 μL to each well of 3 plates to be treated. Add 10 μL sterile YNB to each well of the control plate. Final volume should be 100 μL in all wells.
5. Cover plates with Breathe-Easy membrane (4) and incubate at room temp with shaking at 650 rpm (5) 48 hours.

II-B. Cell dilution and treatment for dose dependent assay

1. In round bottom plate (2) make initial dilution: add 10 μL OD=2 cell culture to 190 μL YNB pH 7.0
2. Use YNB pH 7.0 to dilute cells a second time to 1:10, so that wells are equal to 100 μL, in round bottom 96-well plates (2) (10 μL of OD=0.1 cell culture + 90 μL YNB pH 7.0)
3. Use YNB pH 7.0 to dilute cells a third time to 1:100, so that wells are equal to 100ul, in round bottom 96-well plates (2) (10 μL of 0.01 cell culture + 80 μL YNB pH 7.0)
4. Make caspofungin (A) dilutions of 50 μg/μL, 100 μg/μL, 150 μg/μL, 200u μg/μL and 300 μg/μL in YNB pH 7.
5. Add 10 μL of each corresponding caspofungin dilution concentration to treated rows (note that each column is one strain):
   - 3 untreated rows
   - 1 row each of: 5ug/ul, 10ug/ul, 15ug/ul, 20ug/ul, 30ug/ul
6. Cover plates with Breathe-Easy membrane (4) and incubate at room temp with shaking at 650 rpm 48 hours (5)

III. XTT Analysis

1. Thaw 1 XTT (B) stock tube per treatment plate, and one (or more) 15 μL tube of Menadione (C) stock per set
2. Measure growth (OD_650_) of cells before adding XTT to plates
3. After XTT stock tubes have thawed, add 1 μL menadione (C) to each XTT stock (B) and mix
4. Add 100 μL XTT+Menadione solution to each well, pipetting up and down
   1. apply Breathe Easy membrane (4), cover plates with foil, and incubate at 37°C for 2-3 hours
5. Spin down cells at 3000 rpm (7) for 10min, and transfer 100 μL supernatant into a flat bottom 96 well plate (3)
6. Read plates at OD_490_ on Spectrophotometer (6)

IV. Data interpretation in Excel and GraphPad PRISM

1. Export plate data into excel sheet “XTT data template (96 well format)”
2. Import into PRISM to generate graph of data
   1. Table format: Grouped data, ‘enter __ replicate values in side by side
   2. sub columns
   3. Analyze: all categories, remove baseline and column math
   4. Definition of Baseline: Untreated data set
   5. Calculation: Percent (100*Value/Baseline)
