# Supplementary material for: Membrane Integrity Contributes to Resistance of Cryptococcus neoformans to the Cell Wall Inhibitor Caspofungin
Source: mSphere. 2022 Jun 27;7(4):e00134-22. doi: 10.1128/msphere.00134-22 (PMC9429927; doi:10.1128/msphere.00134-22)
Supplement: TABLE S3 [file msphere.00134-22-s0003.docx]

**Supplemental Table 3:** SDS and caspofungin assay for synergy

| **Strain** | **MIC_50_** | | | | **FICI** |
| --- | --- | --- | --- | --- | --- |
|  | **Alone** | | **Combined** | |  |
|  | **SDS** | **Caspofungin** | **SDS** | **Caspofungin** |  |
| KN99 | 0.015% | 32 ug/mL | 0.009% | 8 ug/mL | 0.25 |
